# Ketorolac plus Lidocaine vs Lidocaine for pain relief following core needle soft tissue biopsy: A CONSORT-compliant double-blind randomized controlled study

**DOI:** 10.1097/MD.0000000000024721

**Published:** 2021-02-19

**Authors:** Thanapon Chobpenthai, Thammasin Ingviya, Pichaya Thanindratarn, Rattakorn Jaiwithee, Kulwadee Sutthivaiyakit

**Affiliations:** a Faculty of Medicine and Public Health, HRH Princess Chulabhorn College of Medical Science, Chulabhorn Royal Academy; b Department of Orthopedics, Chulabhorn Hospital, Bangkok; c Department of Family and Preventive Medicine; d Medical Data Center for Research and Innovation, Faculty of Medicine, Prince of Songkla University, Hat Yai; e Department of Anesthesiology Unit, Chulabhorn Hospital, Bangkok, Thailand.

**Keywords:** ketorolac, Lidocaine, local anesthesia, pain intensity, soft tissue biopsy

## Abstract

**Backgrounds:**

The main objective of this study was to compare the pain control efficacy of local administration of Lidocaine with or without the nonsteroidal anti-inflammatory drug, Ketorolac, and local conventional Lidocaine injection in core needle biopsy of the musculoskeletal tumor.

**Methods:**

The current study was a randomized, double-blind controlled clinical trial that included 128 patients with suspected musculoskeletal tumors. Patients were randomly assigned to either the Ketorolac plus Lidocaine (n = 64) or Lidocaine group (n = 64). The Ketorolac – Lidocaine combination syringe contained 30 mg Ketorolac and 2% Lidocaine – adrenaline dosage, and the Lidocaine syringe contained 2% Lidocaine – adrenaline dosage. The level of pain after core needle biopsy was evaluated for each patient at 1, 6, 12, 24, 48, and >48 hours by a Visual Analog Scale (VAS). The mean VAS changes over time were compared between the Ketorolac plus Lidocaine and Lidocaine groups using a linear mixed model.

**Results:**

baseline information including mean age of patients in Lidocaine group (51.5 ± 19.4 years) and in Lidocaine – Ketorolac combination group (50.1 ± 18 years), diagnosis (malignant, benign, metastatic, infection), tumor location (upper and lower extremities, back), VAS score 1-hour post-operation (mild and moderate pain) were noted. The VAS score ratings were significantly lower in Lidocaine – Ketorolac combination group when compared to the Lidocaine group during the 1 to 24 hours post-operation time period.

**Conclusion:**

Patients receiving Lidocaine – Ketorolac combination dosage had significantly lower VAS scores, and these results confirm that local injection of Lidocaine – Ketorolac combination had a superior pain-controlling effect during the first 24 hours after the biopsy procedure in comparison to Lidocaine injection alone, as measured by VAS score scale.

## 1 Introduction

Core needle biopsy is the most extensively employed biopsy technique for the collection of tissue samples from musculoskeletal tumors and had often been associated with post-procedural pain.^[1]^ Administration of local anesthesia-Lidocaine and adrenaline, a gold standard anesthetic combination, is being widely utilized for post-procedural pain management.^[2,3]^ This local anesthetic effect of Lidocaine and adrenaline persists for a shorter time period of 4 minutes to 1 hour and 30 minutes after injection. Thus, necessitating prolonged pain relief medications. Various pre-, intra-, and post-procedural interventions have been reported to be effective in the management of postoperative pain.^[2]^

Considering the designated duration of pain control effect and significant side effects associated with individual drugs, nonsteroidal anti-inflammatory drugs (NSAID) Ketorolac is established as the best-suited therapeutic regime for managing post-procedural pain after core needle biopsy.^[4–8]^ Ketorolac is considered safe when administered parentally at a maximum dosage of 7 mg/kg, and injection of 30 mg Ketorolac in combination with a local anesthetic (e.g., Lidocaine) had no adverse renal function or gastrointestinal side effects.^[5,6,9–15]^ Further, none of recent/previous literature pertaining to clinical studies has been reported to compare the post-biopsy pain-controlling efficacies of local injection of Lidocaine with and without Ketorolac in patients undergoing soft tissue biopsy. Furthermore, patients have not been prescribed any NSAIDs or any oral analgesic drug after a biopsy.

We hypothesized that local anesthesia (10 mL 2% Lidocaine with adrenaline) in combination with Ketorolac could provide prolonged pain control and relief without compromising on safety issues. The prescribed dosage utilized in this study was Ketorolac 30 mg administration with a 2% Lidocaine to the sampling area for obtaining biopsy involving pain-free procedure.

However, the efficacy of pre-biopsy administration of Ketorolac in combination with a local anesthetic such as Lidocaine for post-procedural pain in cancer patients needs to be established.

Therefore, the present study aimed at comparing the pain control efficacy of local administration of Lidocaine with or without NSAID, Ketorolac, and local conventional Lidocaine injection in core needle biopsy of the musculoskeletal tumor.

## 2 Materials and methods

The clinical trial was a randomized, controlled, double-blind study recruiting patients from the Orthopedic Oncology Surgery Clinic of a Tertiary Care Medical Cancer Center (the HRH Princess Chulabhorn College of Medical Science, Chulabhorn Royal Academy, Bangkok, Thailand).

The study methodology was performed and represented as per CONSORT guidelines. No changes were made to the methodology of study and trial outcomes after trial commencement. The random allocation was generated by the study statistician, and methodology to generate the random allocation sequence was computer-generated. Patients were randomized in a 1:1 ratio. A block size of 4 was used.

The following inclusion and exclusion criteria were considered during the enrollment of patients for the study. Inclusion criteria:(1) Painless soft tissue and bone tumor (including metastasis) with soft tissue extension, tumor-like lesion requiring core needle biopsy; (2) Any age and sex; (3) First time for biopsy; (4) No pain at other sites and needing medication. Exclusion criteria:(1) Soft tissue tumor requiring biopsy by other techniques; (2) Recurrent soft tissue tumor; (3) Weakness or numbness of biopsy area; (4) Not fully consciousness or cooperative; (5) Allergy to NSAIDs; (6) Renal impairment.

All patients had undergone magnetic resonance imaging of the affected part for evaluation of the soft tissue area, together with a core needle biopsy. All the patients received appropriate counseling regarding the study objectives, procedures, possible risks, benefits, and alternatives to participation as well. All procedures performed as part of this study were carried out at the HRH Princess Chulabhorn College of Medical Science, Chulabhorn Royal Academy, Bangkok, Thailand. Informed consent was obtained from each patient for participation in the study.

Patients in the control group received 10 mL of 2% Lidocaine mixed with adrenaline, and the test group received 10 mL of 2% Lidocaine mixed with adrenaline and 30 mg Ketorolac. The following measures ensured blinding: the injectable solutions were formulated by health care personnel (e.g., a nurse or pharmacist) not involved in any other study activity; unlabeled and/or unidentifiable syringes containing concealed injectable solution were used; patients were randomized using a computerized block randomization procedure, and each syringe was randomly assigned a number by the preparer (1 = Lidocaine, 2 = Lidocaine + Ketorolac); all the syringes were covered with white tape to conceal the contents of the syringe; and the contents of the syringe were not revealed to the injector, patient, or evaluator.

**Figure 1:**
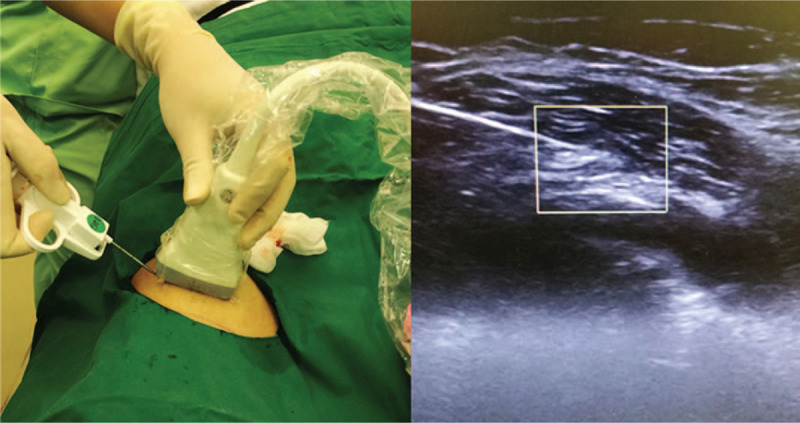
Ultrasound-guided core needle soft tissue biopsy in the leg.

Each patient was asked to complete a Visual Analog Scale (VAS) for evaluating pain before receiving the injection. The biopsy site was prepared using Chlorhexidine, and the core needle biopsy was performed after the respective injection, according to the magnetic resonance imaging evaluation. The core needle was inserted in the tumor-containing soft tissue under ultrasound guidance, once the patient felt numbness in the area (Fig. 1). Each patient was then asked to complete VAS pain assessments at 1, 6, 12, 24, 48, and >48 hours (from 60 to 72 hours) after core needle biopsy. The VAS was classified as mild (score 1–3), moderate (score 4–7), and severe (score 8–10) pain.^[16]^

The VAS score at a single time point is subjective and less informative and we therefore analyzed the mean VAS pain score at different time points as the primary outcome measure. Changes in the VAS score over time were compared between the Ketorolac – Lidocaine combination and Lidocaine groups using a linear mixed model, adjusting for the variability of VAS score within and between patients and for sex, diagnosis, location, and patient age. VAS scores were compared between the 2 groups at different time points after core needle biopsy by multiple linear regression adjusting for sex, diagnosis, location, and patient age. All analyses were performed using R version 3.6.1 (R core team, Austria).

The assistant nurses not involved in drug dosage and administration of medications/ injection enrolled and assigned participants. The assistant nurses not involved during operation procedures were responsible for patient screening and recruitment. Each patient assignment was obtained by drawing prepared numbers from the sealed opaque enveloped by the study statistician.

### 2.1 Statistical analysis

Based on an alpha error of 0.05 and power of 0.8, the required sample size was calculated based on the hypothetical differences in mean VAS scores between the test and control groups, which were 0.3 ± 0.48 and 0.6 ± 0.70, respectively according to the study of K.S. Min et.al. The required sample size was 64 patients per group (10). The mean VAS pain scores for the 2 treatment groups were compared using a linear mixed model adjusting for random effects of time after surgery, age, sex, location of the tumor, and primary diagnosis, with a significance level of .05.

## 3 Results

A total of 128 patients who met the inclusion criteria were enrolled in this study. Of these 128 patients, 64 were randomly assigned to the Lidocaine group and 64 patients to the Lidocaine – Ketorolac combination group. The mean age of patients in the Lidocaine group was 51.5 ± 19.4 years, and in Lidocaine – Ketorolac combination group, it was 50.1 ± 18 years. Information related to gender, diagnosis (malignant, benign, metastatic, infection), tumor location (upper and lower extremities, back), VAS score 1-hour post-operation (mild and moderate pain) for Lidocaine group and Lidocaine – Ketorolac combination group are represented in baseline information Table 1. All patients completed the study successfully, and none of the patients were lost during the follow-up period.

**Table 1:** Patient base line characteristics.

	Lidocaine	Lidocaine + ketorolac
N	64	64
Age (yr)		
Mean	51.5	50.1
Standard deviation	19.4	18
Sex		
Male	26 (40.6%)	23 (35.9%)
Female	38 (59.4%)	41 (64.1%)
Diagnosis		
Malignant	22 (34.4%)	20 (31.2%)
Benign	29 (45.3%)	29 (45.3%)
Metastasis	13 (20.3%)	13 (20.3%)
Infection	2 (3.1%)	2 (3.1%)
Tumor location		
Upper extremities	22 (34.4%)	31 (48.4%)
Lower extremities	36 (56.2%)	24 (37.5%)
Back	6 (9.4%)	9 (14.1%)
VAS score 1-h post-operation		
Mild pain (1–3)	56 (87.5%)	64 (96.9%)
Moderate pain (4–6)	8 (12.5%)	2 (3.1%)

**Figure 2:**
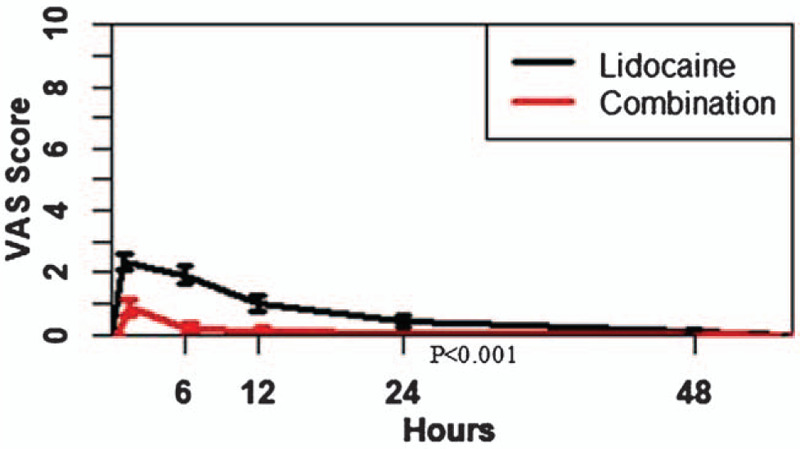
Mean VAS after core needle biopsy at 0, 1, 6, 12, 24, 48, and >48 h post-operation.

Patients in both groups experienced pain relief (decreased pain after the procedure) at 1, 12, 24, 48, and >48 hours after a core needle biopsy, as evidenced by the VAS pain scores recorded at respective time points. Patients in the Lidocaine – Ketorolac combination group exhibited significantly greater improvements in VAS pain scores from 1 to 24 hours after the biopsy, in comparison to patients in the Lidocaine group (*P* < .001) (Fig. 2; Table 2).

**Table 2:** Results of linear mixed and linear model adjusted for sex, diagnosis, location, and age.

	VAS score		
Time	Lidocaine	Lidocaine + NSAID	*P* value
Overall	0.70 (0.17)^∗^	0.26 (0.14)^∗^	<.001^∗^
Subgroup by time (h)			
0	0	0	
1	2.36 (1.03)	0.86 (1.05)	<.001^∗∗^
6	1.92 (1.18)	0.19 (0.59)	<.001^∗∗^
12	1.00 (0.93)	0.12 (0.45)	<.001^∗∗^
24	0.44 (0.71)	0.03 (0.17)	<.001^∗∗^
48	0.09 (0.29)	0.02 (0.12)	.04^∗∗^
>48	0 (0)	0 (0)	NA

One patient in the Lidocaine group experienced a fainting episode, secondary to a vasovagal reaction, but this resolved within 5 minutes of the occurrence. Further, the patient did not require any medical intervention for this complication. The Lidocaine – Ketorolac combination group was considered to be well tolerated, with no other reported adverse events or complications during the study period.

## 4 Discussion

Patients receiving Lidocaine – Ketorolac combination dosage had significantly lower VAS scores when compared to patients with Lidocaine dosage during a time period of 1 to 24 hours post-operation. These results confirm that the local injection of Lidocaine – Ketorolac combination had a superior pain-controlling effect during the first 24 hours after the biopsy procedure in comparison to Lidocaine injection alone, as measured by the VAS score scale. This prolonged pain control effect can be attributed to the longer half-life (5 hours) of Ketorolac in comparison to only 1.5 hours for Lidocaine, clearly indicating that Lidocaine is eliminated rapidly from the body and hence, had a short-lived pain-relieving effect. In contrast, Ketorolac's longer half-life might have a beneficial effect in prolonging the anti-inflammatory and analgesic actions.^[13,17]^ Therefore, this combination of ketorolac with Lidocaine as local anesthesia is a viable and effective option for patients undergoing soft tissue biopsies.

Ketorolac is currently the only NSAID regarded as safe for intravenous administration. It has both anti-inflammatory, analgesic properties and can be prescribed for a maximum of 5 days.^[4,6,15,18]^ Few trials had reported that Ketorolac is associated with milder gastrointestinal complications compared with other NSAIDs, but others have different opinions.^[4,15,18,19]^

No clinical studies have yet compared the post-biopsy pain-controlling efficacies of local injection of Lidocaine with and without Ketorolac in patients undergoing soft tissue biopsy. However, findings similar to the current results have previously been reported in different clinical settings. Ranjan et al compared the efficacy of 0.75% Ropivacaine with 2% Lidocaine in combination with adrenaline for controlling post-procedural pain after mandibular tooth extraction and found no clear advantage of Ropivacaine over the gold standard Lidocaine – adrenaline combination mixture.^[3]^ Similarly, the addition of Bupivacaine to Lidocaine plus adrenaline showed no benefit compared with the gold standard for controlling pain after eyelid surgery.^[20]^

Seyfi et al reported that the addition of ketorolac to Lidocaine for regional anesthesia significantly reduced the intensity and duration of intraoperative and postoperative pain for up to 24 hours.^[19]^ David et al also reported that the use of ketorolac, Lidocaine, and a combination of the 2 drugs produced superior pain control compared with no intervention, based on postoperative morphine use.^[21]^ Connelly et al reported that 26% of patients achieved complete resolution, and 69% of patients achieved partial relief of sympathetically mediated pain symptoms after intravenous regional anesthetic (IVRA) block containing Ketorolac and Lidocaine.^[22]^ Kao et al developed Lidocaine – Ketorolac combination -loaded nanofibrous anti-adhesion membranes that offered sustained surgical-wound-related pain relief in rats.^[1]^ Jankovic et al reported a significantly lower pain score following Lidocaine – Ketorolac combination and Lidocaine – Ketorolac combination with dexamethasone-induced IVRA compared with Lidocaine alone in patients undergoing ambulatory hand surgery.^[23]^ El-Feky et al developed polymeric wafers that co-delivered Ketorolac and Lidocaine to soft tissues, and noted observations were improved wound healing and pain control compared with a marketed product following gingivectomy.^[24]^ Reuben et al reported that patients who received a combination of ketorolac with Lidocaine for IVRA experienced significantly less intraoperative tourniquet pain and postoperative pain with less requirement for postoperative analgesic tablets.^[25]^ A group of Korean researchers reported that both Paracetamol and Ketorolac, when administered with Lidocaine for IVRA, effectively reduced postoperative pain and the consumption of analgesic tablets post-surgery.^[26]^ Alam et al studied different volumes of Lidocaine typically used for facial skin cancer excision and reconstruction and found that the volumes of Lidocaine used by different surgeons were within the allowed limits and that the toxicity of Lidocaine was rare or absent.^[27]^

The following are the limitations in the current study: Firstly, the enrolled participants were from a single medical center with a relatively small population and sample size. Secondly, the results of our trial cannot be applied to those patients who have contraindications to the medications administered. The VAS shows good reliability for assessing acute pain.^[28]^ However, the VAS has the major drawback of requiring adequate levels of visual acuteness, motor function, and the cognitive ability to translate a sensation of pain into a distance measure.^[18]^ As per the results, VAS score reduction in the value of at least 2 or 30% reduction has been suggested as representing meaningful pain relief to patients that is a significant difference and decrease of VAS score from 3 to 1 can categorize to “much improve”.^[14,29,30]^ The observation period was limited to 1 week with VAS pain scores only recorded at 1, 6, 12, 24, 48, and >48 hours after core needle biopsy. The patients had different types of tumors at different sites, and this variability might have affected the VAS pain scores recorded for different patients after the biopsy. In addition, we only used a single dose of Ketorolac, and further studies are needed to determine if a lower dose can produce similar pain control or if a higher dose can impart better pain control. Furthermore, we did not investigate the effects of systemic administration of Ketorolac, which may provide better pain control. Further large studies are therefore required to validate the efficacy of Ketorolac for prolonging pain control after core needle soft tissue biopsy.

Our clinical trial study exhibited the following strengths: trial was well-designed, prospective, and was a controlled study. Secondly, we adopted the reliable VAS scoring to assess pain intensity. Thirdly, we assessed pre-biopsy pain during 1, 6, 12, 24, 48, and >48 hours after core needle biopsy. Further, for commonly studied post procedures to assess pain, there was a high incidence of pain immediately after post procedures.

## 5 Conclusion

Local injection of 30 mg Ketorolac in combination with Lidocaine resulted in improved pain control compared to Lidocaine alone for up to 24 hours after core needle biopsy in patients with musculoskeletal tumors. Herein, in our study, we used a single dose of Ketorolac. However, further studies are needed to examine the efficacy of a lower dose with similar pain control management or even a higher dose to examine better pain control management. Additionally, in our study, we did not investigate the effects of systemic administration of Ketorolac, which may provide a better pain control strategy. Further, larger clinical studies are required to validate the efficacy of Ketorolac for prolonging pain control management after core needle soft tissue biopsy.

## Acknowledgments

We thank Susan Furness, PhD, from Edanz Group (https://en-author-services.edanzgroup.com/) for English editing a draft of this manuscript. We also thank Piya Kiatisevi, MD for inspiring us to perform this research with the goal of improving patients’ quality of life.

## Author contributions

**Conceptualization:** Thanapon Chobpenthai, Pichaya Thanindratarn, Rattakorn Jaiwithee, Kulwadee Sutthivaiyakit.

**Data curation:** Thanapon Chobpenthai, Rattakorn Jaiwithee.

**Formal analysis:** Thanapon Chobpenthai, Thammasin Ingviya, Pichaya Thanindratarn.

**Investigation:** Thanapon Chobpenthai.

**Methodology:** Thanapon Chobpenthai, Thammasin Ingviya, Kulwadee Sutthivaiyakit.

**Project administration:** Thanapon Chobpenthai.

**Resources:** Thanapon Chobpenthai.

**Supervision:** Thanapon Chobpenthai.

**Validation:** Thanapon Chobpenthai, Thammasin Ingviya.

**Visualization:** Thanapon Chobpenthai, Thammasin Ingviya.

**Writing – original draft:** Thanapon Chobpenthai.

**Writing – review & editing:** Thanapon Chobpenthai, Thammasin Ingviya, Pichaya Thanindratarn, Rattakorn Jaiwithee, Kulwadee Sutthivaiyakit.
